# Varicella Pneumonia in Otherwise Healthy 31-Year-Old Female Nonsmoker With No Prior Varicella Immunity

**DOI:** 10.7759/cureus.40507

**Published:** 2023-06-16

**Authors:** Michal Kawzowicz, Maha Bayya

**Affiliations:** 1 Internal Medicine, Upstate University Hospital, Syracuse, USA

**Keywords:** varicella-zoster virus infection, varicella radiograph, varicella zoster (chicken pox), varicella-zoster virus, varicella pneumonia

## Abstract

Varicella pneumonia is a relatively rare but severe complication of primary varicella infection. It primarily affects adults and is exacerbated by risk factors such as smoking, immunocompromised status, and male gender. Left untreated, the mortality associated with varicella pneumonia is high and ranges from 10-30 percent in the literature. This is a case of an adult female patient with no prior immunity to varicella who presented with increased oxygen requirements approximately 5 days after the onset of cutaneous symptoms. Her symptoms quickly improved after 1-day post administration of valacyclovir, and on discharge, she was prescribed a 7-day course of valacyclovir. Early treatment with acyclovir is the mainstay of treatment and is essential as it has been shown to reduce long-term complications and decrease mortality. Despite the low incidence of varicella pneumonia among patients with primary varicella infection, the index of suspicion for varicella pneumonia among patients with primary varicella infection should be high in the setting of increased oxygen requirements due to the mortality and symptomatic benefits of early treatment.

## Introduction

The course of primary varicella infection is typically benign in the pediatric population, however, the chance of complications is approximately 25 times higher in adults [1]. The onset of varicella pneumonia occurs typically 1-6 days after cutaneous symptoms and is most commonly associated with dyspnea, pleuritic chest pain, cough, tachypnea, and fever. The probability of onset of varicella pneumonia is approximately 1 in 400 for healthy adults with the onset of chickenpox. Risk factors for varicella pneumonia include smoking, male gender, immunosuppression, and adult onset of primary varicella infection. Varicella pneumonia is one of the most serious complications of adult-onset primary varicella infection, and symptomatic relief along with decreased mortality is associated with early treatment with acyclovir [2].

## Case presentation

**Figure 1:**
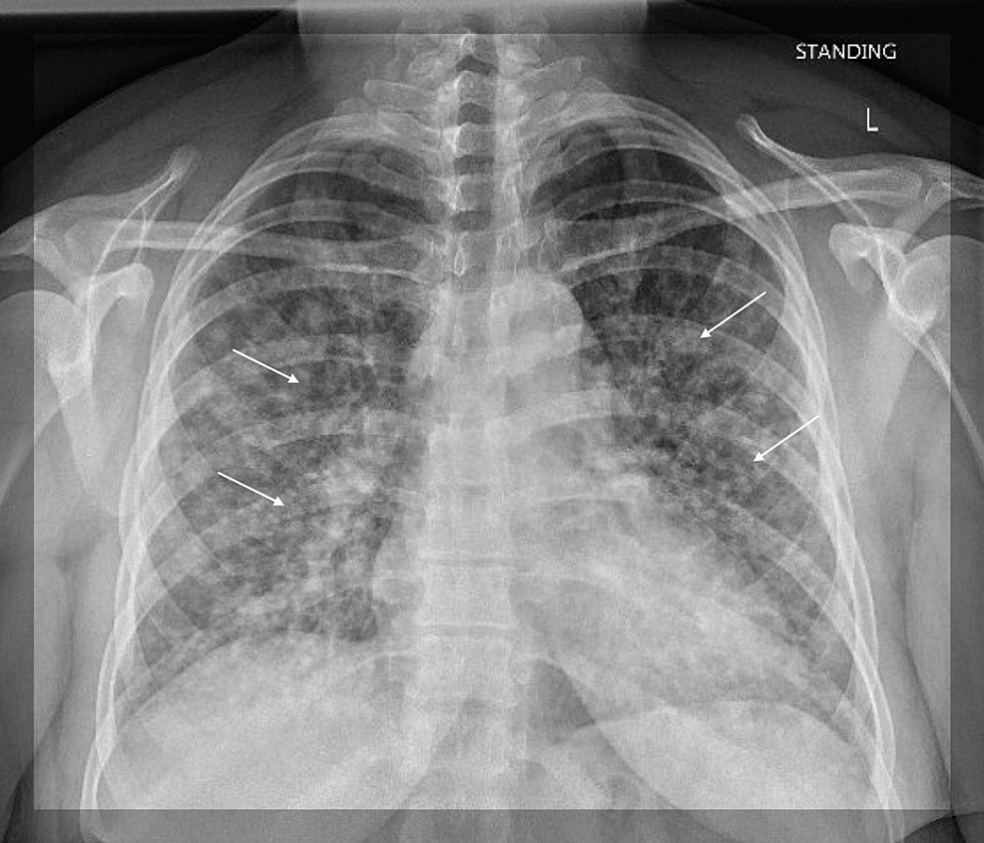
Chest x-ray of patient on admission Chest x-ray on the day of admission showing diffuse nodular and interstitial lung infiltrates characteristic of varicella pneumonia

**Figure 2:**
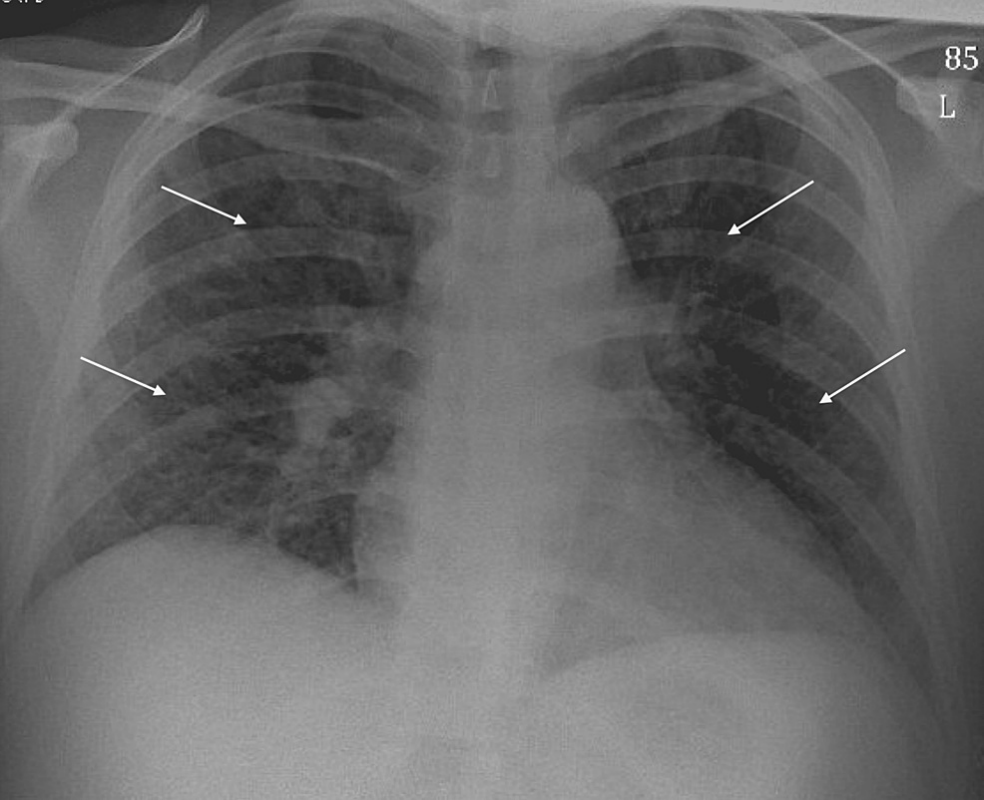
Chest x-ray of patient on day prior to admission Chest x-ray on the day prior to admission showing no lung consolidations and grossly unremarkable findings

**Figure 3:**
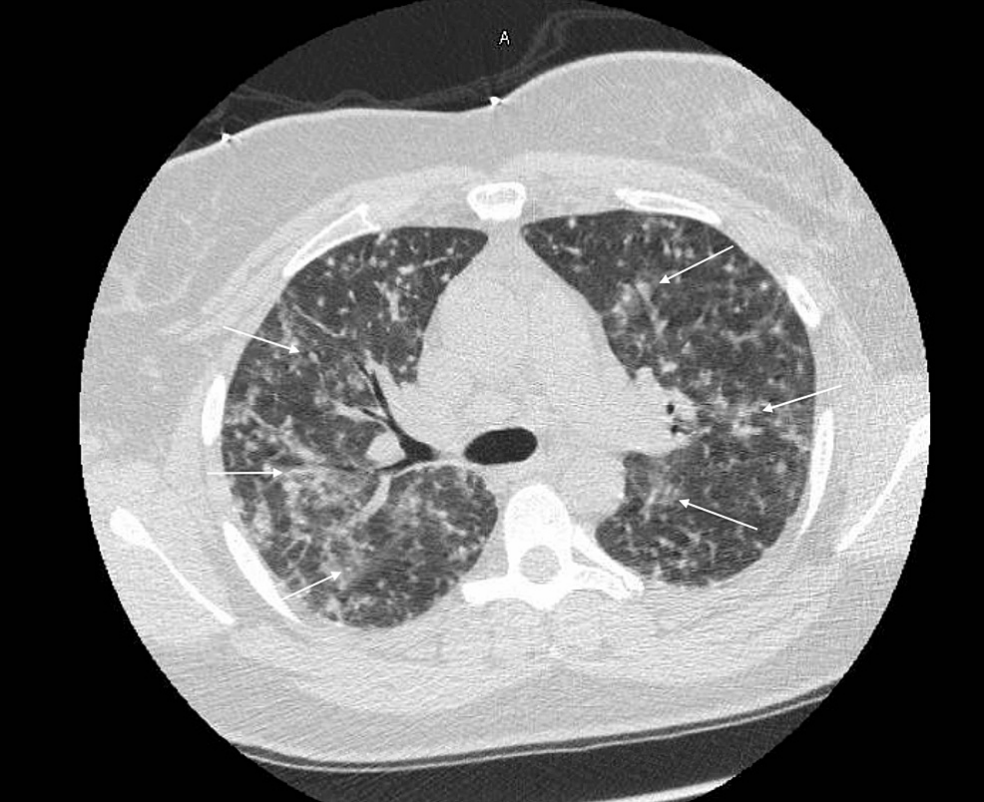
Axial high-resolution CT image of patient on admission Axial high-resolution CT image on the day of admission showing diffuse micronodular lung infiltrates characteristic of varicella pneumonia

A 31-year-old female patient presented to the emergency room with approximately 1 week of shortness of breath which has progressively worsened prior to admission. The patient presented to the Emergency Department 3 days prior to admission for fever and headache along with new-onset rash, but no requirements for supplemental oxygen, and was subsequently discharged with conservative measures. The patient was exposed to varicella from her husband who experienced a recent onset of shingles. The patient reported that she has not received the varicella vaccine, and never experienced varicella infection in the past. The lack of prior immunity was her only significant risk factor, as she was immunocompetent and had no history of smoking. Her clinical presentation included painful and itchy maculopapular rash most prominent on the face but also present scattered throughout her body. The patient’s white blood cell count was 4.1 on admission with an absolute lymphocyte count of 0.8. Chest x-ray on admission showed diffuse bilateral interstitial infiltrates (Figure 1) and was not present on a chest x-ray taken the day prior which showed grossly unremarkable lung findings (Figure 2). CT Thorax while inpatient showed a diffuse micronodular pattern distributed throughout the lungs (Figure 3). 

 

The patient was provisionally diagnosed with varicella pneumonia on admission. The inpatient Infectious Disease service was consulted and agreed with the provisional diagnosis of varicella pneumonia. The patient was placed on a 7-day course of valacyclovir. On the first day of admission, the patient required supplemental oxygen and was on a 2L nasal cannula (the patient was not on oxygen at home), and any attempts to titrate oxygen down resulted in shortness of breath. On the second day of the patient’s inpatient course, she reported a significant improvement in respiratory and cutaneous symptoms and was successfully weaned off supplemental oxygen. The prominence of cutaneous symptoms including skin rash and itchiness has decreased noticeably. The patient was subsequently cleared for discharge from the Infectious Disease and General Medicine services and was instructed to complete her valacyclovir course outpatient.

## Discussion

Adult onset of primary varicella infection is typically instigated by close proximity and airborne transmission with an infectious carrier [3]. Entry of the virus may occur through the patient’s respiratory system or conjunctiva [1]. Post-infection viremia with the varicella-zoster virus occurs within the patient’s lymph nodes and subsequently spreads to other organ systems including the lungs, bone marrow, pancreas, and liver with viral replication occurring mainly within macrophages [1]. While most cases of varicella occur in children and the clinical course of approximately 1 week is typically benign [3,4], adult populations with the onset of chickenpox typically experience complications at a rate of approximately 25 times the pediatric age group [1]. Complications may include neuralgia, cellulitis secondary to open sores, encephalitis, and pneumonia [3,5]. One of the most serious complications associated with adult chickenpox is varicella pneumonia. The onset of varicella pneumonia in adults is estimated to occur in approximately 1 out of every 400 chickenpox cases [1] and is associated with an overall mortality rate ranging from 10-30 percent without treatment [3]. Risk factors for contracting varicella pneumonia include smoking, pregnancy, immunocompromised patients, and male gender [6]. These risk factors are also associated with less favorable long-term outcomes and increased mortality [1]. 

The onset of varicella pneumonia is often gradual and progressive, typically occurring within 1-6 days of cutaneous symptoms, and typically presents as dyspnea, tachypnea, and dry cough [6]. Cases have been reported of pulmonary symptoms occurring prior to the onset of rash [1]. Radiologic findings include the presence of ground glass attenuation of lung parenchyma, pulmonary nodules, and nodular coalescence. These findings typically resolve after the resolution of cutaneous symptoms [7]. The prominence of radiological findings was found to correlate with the severity of respiratory symptoms [3]. The first line treatment for varicella pneumonia is acyclovir for a 7-10 day course [2]. The respiratory and cutaneous symptoms typically resolve within several days of starting acyclovir therapy [3]. With early treatment, the most likely outcome of varicella pneumonia is complete resolution [3], and delay or failure to administer treatment is associated with increased mortality [1,2].

## Conclusions

Varicella pneumonia is uncommon in patients with primary varicella infection but is associated with high mortality. A history of smoking, male gender, and immunocompromised status are among the risk factors that increase the severity of the disease course and increase the probability of mortality. If untreated, the mortality associated with varicella pneumonia has been reported to be as high as 30 percent in some studies. Acyclovir is the first-line treatment of varicella pneumonia. Radiologic findings characteristic of varicella pneumonia include ground-glass attenuation of lung parenchyma on x-ray and diffuse micronodular appearance on high-resolution CT Thorax. Varicella pneumonia should be suspected in the setting of increased oxygen requirements in the setting of primary varicella infection and treatment with acyclovir should be initiated quickly as early treatment is associated with a significant reduction in mortality and morbidity.
